# Retraction Note: Epstein–Barr virus-encoded microRNA BART1 induces tumour metastasis by regulating PTEN-dependent pathways in nasopharyngeal carcinoma

**DOI:** 10.1038/s41467-025-65152-w

**Published:** 2025-10-14

**Authors:** Longmei Cai, Yanfen Ye, Qiang Jiang, Yuxiang Chen, Xiaoming Lyu, Jinbang Li, Shuang Wang, Tengfei Liu, Hongbing Cai, Kaitai Yao, Ji-Liang Li, Xin Li

**Affiliations:** 1 Cancer Research Institute, Southern Medical University, Guangzhou, 510515 China; 2 Radiation Department, The First Affiliated Hospital, Wannan Medical College, Wuhu, 241001 China; 3 Department of Pathology, Basic Medical College, Southern Medical University, Guangzhou, 510515 China; 4 School of Traditional Chinese Medicine, Southern Medical University, Guangzhou, 510515 China; 5 School of Biotechnology, Southern Medical University, Guangzhou, 510515 China; 6 Molecular Oncology Laboratories, Department of Oncology, Weatherall Institute of Molecular Medicine, University of Oxford, John Radcliffe Hospital, Oxford, OX3 9DS UK

Retraction to: *Nature Communications* 10.1038/ncomms8353, published online 2 July 2015

The Editors have retracted this article. After publication, concerns were raised regarding image overlap in Fig. 7a, which the authors addressed by a Correction^1^. However, further concerns were subsequently identified:

- Fig. 2a CNE-1 Mock and Fig. 2c 5-8F-BART1 in-b1-5p images appear to overlap.
- Fig. 6b 5-8F-BART1 PTEN image appears to overlap with Fig. 7a in-NC (and the corresponding image in the Correction [1] Supplementary Fig. 1);
- Fig. 7b in-b1-3p image appears to overlap with the corrected Fig. 7a in-b1-3p (and the corresponding image in the Correction [1] Supplementary Fig. 1);
- In the full western blot images in Fig. S9, Fig. 4a CNE1 p-ERK1/2 and Fig. 4b CNE1 p-Akt blots appear to share the same background, although the marker size is labelled differently.
- Correction [1] Supplementary Fig. 1 in-NC set 2 and 3 images appear to overlap;
- The CNE1, 5-8F and HONE1 cell lines used in this study have been reported to be contaminated with HeLa cervical cancer cells.

The Editors therefore no longer have confidence in the presented data and the conclusions of this article.

Xin Li has stated on behalf of all authors that they agree with this retraction.
